# Effect of salinity on growth and microbial diversity in cultures of *Scenedesmus almeriensis* produced at a pilot scale

**DOI:** 10.3389/fbioe.2026.1753183

**Published:** 2026-02-23

**Authors:** Elia Suyapa Rivera-Sánchez, María Salinas-García, Emanuele Viviano, Silvia Villaró-Cos, Tomás Lafarga

**Affiliations:** 1 Department of Chemical Engineering, University of Almeria, Almeria, Spain; 2 Faculty of Science, Universidad Nacional de Agricultura, Catacamas, Honduras; 3 CIESOL Solar Energy Research Centre, Joint Centre University of Almeria-CIEMAT, Almeria, Spain

**Keywords:** bioeconomy, metagenomics, microalgae, microbial contaminants, photobioreactor, renewable biomass

## Abstract

**Introduction:** Freshwater scarcity represents a major constraint for the sustainable industrial-scale cultivation of microalgae. This study investigates the feasibility of producing *Scenedesmus almeriensis* using seawater in 3.1 m^3^ tubular photobioreactors under winter-spring conditions. The appearance of algal predators represents a significant challenge in industrial facilities, and this research also explores whether seawater can serve as a strategic water source for more resilient and efficient production systems.

**Methods:** Biomass productivity and microbial diversity were compared between freshwater and seawater-based cultures under batch and semi-continuous regimes at dilution rates of 0.1, 0.2, and 0.3 day^-1^. The production was carried out in duplicate using identical tubular photobioreactors. Analytical determinations included measuring biomass concentration, chlorophyll fluorescence, and oxygen production via photorespirometry. Microbial diversity was assessed through microscopy and metagenomic analysis (18S and 16S rDNA) to identify taxonomic classifications and potential biotic contaminants.

**Results and Discussion:** Maximum biomass concentrations reached 0.60 and 2.15 g·L^-1^ in freshwater and seawater, respectively. Production using seawater led to a higher biomass productivity (0.18 g·L^-1^·day^-1^) compared to freshwater (0.06 g·L^-1^·day^-1^) at a fixed dilution rate of 0.1 day^-1^. Seawater cultures exhibited greater stability and higher photosynthetic efficiency, with *Scenedesmus* dominating up to 70% of the microalgal community due to reduced contamination by zooplankton, fungi, and ciliates. In contrast, freshwater cultures were rapidly degraded by rotifers and anaerobic fungi, leading to a culture crash when dilution rates were increased. These findings highlight the potential of seawater to act as a biological barrier against contaminants while significantly reducing freshwater requirements in industrial microalgae production.

## 1 Introduction

The production of microalgae has become increasingly important due to its potential as a sustainable alternative in a variety of industries including food, cosmetics, pharmaceutical, water treatment, and agricultural sectors (Lafarga and Acién, 2022). By decoupling biomass production from arable land, microalgae offer a pathway to reduce the ecological footprint of industrial commodities (Ben Ouada and Ammar, 2024). However, the transition from laboratory success to industrial-scale profitability remains hindered by significant technological and economic bottlenecks, primarily regarding resource intensity and biological stability (i.e., growth of algal predators or unwanted microalgae).

Although microalgae are diverse, only a few are industrially relevant. These include *Arthrospira platensis*, *Dunaliella salina*, *Haematococcus pluvialis,* and *Tetraselmis chuii* (Morillas-España et al., 2022)*.* One of the main reasons for their commercial success is that they are extremophiles and their production is somehow easier due to the lower appearance of biotic contaminants. For example, *Arthrospira platensis* blooms in alkaline lakes with high pH in the range 9–11 and *Dunaliella salina* grow well on a high salinity up to 3 M (Varshney et al., 2015). Species of the genus *Scenedesmus* have shown a high potential for industrial applications due to their rapid growth and their ability to adapt to stressful environmental conditions (Morillas-España et al., 2020). Some of these species have demonstrated biostimulant effects (Rivera-Sánchez et al., 2025) or are rich sources of carotenoids with commercial interest (Granado-Lorencio et al., 2009). Despite being robust and tolerate a wide range of environmental conditions, their optimal growth conditions are generally achieved at a mild alkaline pH and using freshwater. These conditions facilitate the growth of unwanted contaminants that can include rotifers, bacteria, or other algae. It has been suggested that producing microalgae inland using seawater might reduce the risk of biotic contamination. To date, this strategy has not been attempted using strains belonging to the *Scenedesmus* genus.

In addition to limiting the growth of unwanted microorganisms, the use of seawater might also contribute to a constraint often overlooked in sustainability metrics that is freshwater consumption. Freshwater is an increasingly limited resource. It is estimated that approximately 1,000 L of water are required to produce just 1 kg of microalgae biomass (Murphy and Allen, 2011). Despite being lower than that of other animal- and plant-derived products rich in proteins, the production of microalgae using seawater shows potential to further reduce the freshwater requirements. In this context, *Scenedesmus almeriensis production* could also benefit from the benefits of producing using of seawater. It is important to highlight that shifting freshwater strains to saline media introduces physiological trade-offs. The metabolic energy diverted toward osmoregulation and ion pumping can reduce overall biomass productivity and alter the composition of the cells. These phenomena occurred previously when producing *Arthrospira platensis* (Spirulina) using seawater after an acclimatation stage (Villaró et al., 2023b).

In this context, the main objective of this study was to evaluate the potential production of *Scenedesmus almeriensis* using only seawater. This study also aimed at evaluating the effect of salinity on growth and on the microbial diversity of the culture, which is generally overlooked. The biomass production was carried out using 3.1 m^3^ tubular photobioreactors located inside a greenhouse during winter/spring.

## 2 Materials and methods

### 2.1 Microalgae used and composition of the medium

The inocula of *Scenedesmus almeriensis* CCAP 276/24 used in this study were prepared using 5 L controlled bubble column photobioreactors maintained at 23 °C, pH 8.0 and 500 μmol m^−2^ s^−1^ photons and 100 L bubble columns with a controlled pH of 8.0. The bubble columns were located inside the same greenhouse than the tubular photobioreactors described below. The culture medium used for the microalgae production was described elsewhere (Morillas-España et al., 2020). Two media were prepared, one using freshwater and another one using seawater, collected directly from the Mediterranean Sea (36°49′40.7′'N, 2°24′08.2′'W). The microalga was acclimated to seawater as described elsewhere (Rivera-Sánchez et al., 2025).

### 2.2 Photobioreactors used

**FIGURE 1:**
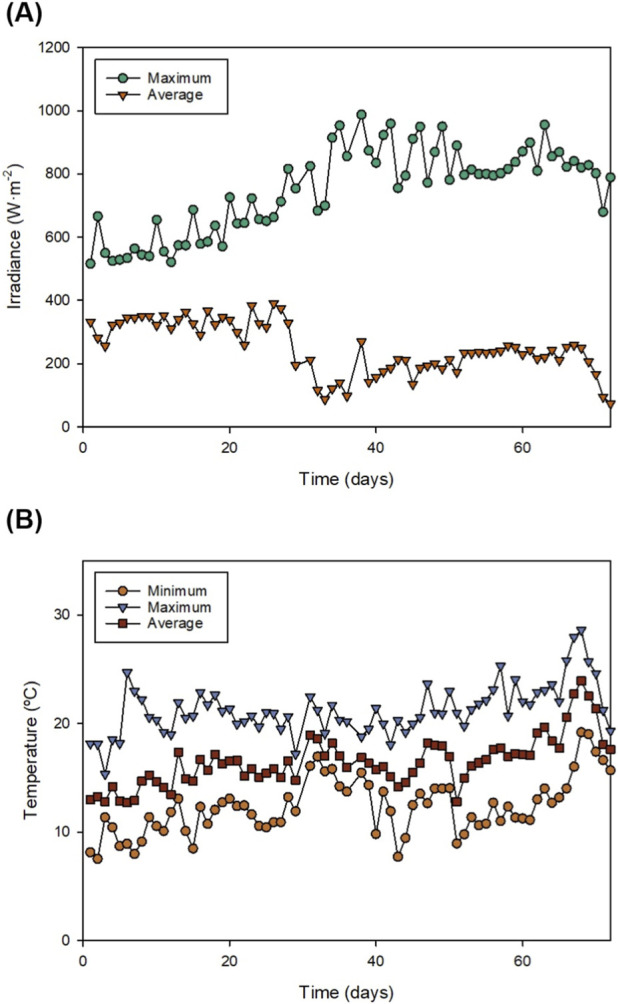
Environmental conditions inside the greenhouse during biomass production using 3.1 m^3^ tubular reactors: **(A)** Irradiance reaching the surface of the culture and **(B)** temperature.

Biomass production was carried out in duplicate in winter-spring using two identical 3.1 m^3^ tubular photobioreactors located at the CIESOL microalgae demonstration plant (36°50′03.3″N 2°24′09.8″W). The CIESOL microalgae demonstration plant is equipped with sensors that are continuously monitoring environmental and culture conditions (e.g., irradiance, temperature, humidity, pH). Each tubular photobioreactors includes a 0.7 m^3^ bubble column that stands 2.30 m tall with a diameter of 0.64 m. It is used for mixing (carbon dioxide injection) and degassing the culture, with dissolved oxygen removed by a constant airflow of around 150 L min^-1^. The photobioreactor’s tubing system is 28 m long, with a tube diameter of 0.09 m, arranged in seven parallel conduits on each side. These tubes, fabricated from methacrylate and joined in a loop, have an approximate combined volume of 2.4 m^3^ and span a total horizontal length of about 400 m. All the reactors were identical and operated under the same environmental conditions (Figure 1). Biomass production was started in batch mode until the stationary phase was reached; at this point, the production mode was switched to semi-continuous mode with a dilution rate of 0.1–0.3 days^-1^. Semi-continuous production was maintained for approximately 70 days. Temperature, pH and dissolved oxygen were monitored using commercial probes connected to an MM44 transmitter controller (Crison Instruments, Barcelona, Spain) and LabVIEW data acquisition software (National Instruments, Austin, TX, United States). The pH was kept constant at 8.0 ± 0.1 by injecting pure carbon dioxide. The biomass was harvested by centrifugation, frozen and freeze-dried until further use.

### 2.3 Analytical determinations

The biomass concentration, expressed as dry weight, and the chlorophyll fluorescence ratio (Fv/Fm) were measured as described in previous studies (Villaró et al., 2023a). The concentration of N-NO_3_^-^ and P-PO_4_^3-^ was determined following standard procedures (Ciardi et al., 2022). All the determinations were made daily and in triplicate per natural replicate.

To assess the effect of seawater on the morphology of the cells, the cultures were also analysed by field emission scanning electron microscopy (FESEM). The samples for FESEM analysis were collected at day 25, at the end of the batch phase, and dialysed (SnakeSkin™ 3,500 MWCO, Thermo Scientific, IL, United States) against distilled water overnight to remove salts. FESEM measurements were conducted using a Zeiss Sigma 300 VP field emission scanning electron microscope with an attached energy-dispersive X-ray analyser (Oxford Instruments). The cultures were covered by a thin layer of conductive carbon (5–10 nm) under vacuum conditions (10^–7^ mbar). Magnification ranged from 500 to 60,000 X, and the accelerating voltage was set at 5–10 kV.

### 2.4 Photorespirometry

Photorespirometry analyses were performed following the protocol described elsewhere (Sánchez-Zurano et al., 2020). Briefly, a 100 mL jacketed cylindrical reactor was used containing 80 mL of culture was used. Agitation (150 rpm), temperature (25 °C), and pH (8.0) were controlled. The pH was controlled using pure carbon dioxide. The irradiance was measured using a spherical quantum sensor (SQS-100; Walz GmbH, Effeltrich, Germany). The protocol followed consisted of alternating 4 min light–dark cycles to quantify oxygen production during photosynthesis in the light phase and oxygen consumption (respiration) in the dark phase. From these measurements, the net oxygen production rate (OPR), defined as the amount of oxygen produced in the light phase minus the amount of oxygen consumed in the dark phase, was calculated. The OPR was determined in triplicate per natural replicate and the results were expressed as mg_O2_·g^-1^·h^-1^.

### 2.5 Metabolomic analyses

The genetic identification of the strain was conducted by Stab Vida, Lda. (Lisbon, Portugal) to confirm its taxonomy classification. Briefly, the analysis employed the molecular marker 18S rDNA - V9 (V8F and 1510R), a widely used target in phylogenetic studies of Chlorophyta and 16S V3-V4 (16S_F (341F) and 16S_R (785R)) for the identification of prokaryotic microorganisms. The genomic DNA extraction was performed using the Maxwell^®^ RSC PureFood GMO and Authentication Kit (Promega Biotech Ibérica SL, Madrid, Spain), ensuring high-quality DNA for sequencing and downstream bioinformatic analyses. Sequencing was performed on the Illumina MiSeq platform with the MiSeq Reagent Kit v3, generating 300 bp paired-end reads. The raw sequence data were processed and analyzed using QIIME2 (2024.2 version), a robust bioinformatics pipeline for quality control, sequence alignment, and taxonomic classification. The samples sequenced were collected at day 25, at the end of the batch phase. They were then frozen and stored at −80 °C until analysis.

### 2.6 Statistical analysis

All experiments were performed using identical 3.1 m^3^ tubular photobioreactors as natural replicates. For each reactor, analytical determinations were performed daily and in triplicate. Results are expressed as the mean ± standard deviation (SD). Significant differences between the freshwater and seawater cultures at various dilution rates were determined using a one-way analysis of variance (ANOVA) followed by Tukey's post-hoc test. In all cases, the level of statistical significance was set at *p <* 0.05 and the analyses were done using Statgraphics Centurion v19 (Stat-graphics Technologies Inc., VA, United States).

## 3 Results and discussion

### 3.1 Biomass productivity

The environmental conditions within the greenhouse were monitored throughout the experimental period, as light availability is a primary determinant of photosynthetic efficiency and biomass yield. The performance of the culture was linked to the solar energy available within the greenhouse. Figure 1A shows the irradiance reaching the surface of the tubular photobioreactors. The maximum irradiance increased from 550–650 W m^-2^ during the first 10 days to approximately 1000 W m^-2^ between days 30 and 45 and then stabilised at around 800–900 W m^-2^ for the rest of the production stage. The average irradiance reaching the culture, which is a more relevant measurement, ranged between 300–350 W m^-2^ during the first half of the experiment, and around 200 W m^-2^ during the last weeks of biomass production. Although very high irradiances can cause photooxidation and stress (Pires et al., 2014), moderate levels favour cell aggregation and optimise nutrient uptake. In fact, a previous study showed that an average daily irradiance of approximately 259 W m^-2^, combined with optimal temperature, maximised biomass productivity (Arcila and Buitrón, 2017). *Scenedesmus almeriensis* is a robust strain that can cope the irradiances achieved in this study without photoinhibition (Cerdá-Moreno et al., 2024).

Like irradiance, temperature (Figure 1B) plays a crucial role in microalgae growth. Temperature influences cell composition, nutrient uptake, CO_2_ fixation and growth rates (Singh and Singh, 2015). Overall, the maximum culture temperatures reached during the biomass production were around 20 °C–25 °C. The average daily temperature was generally around 15 °C–20 °C, which is slightly lower than the optimal growth temperature (20 °C–30 °C) for many microalgae (Dalirynezhad et al., 2018). In the case of *Scenedesmus*, temperatures around 35 °C have shown to increase biomass production by 26% compared to 25 °C (Feng et al., 2024). However, such high temperatures might cause a damage to the photosynthetic apparatus depending on the duration of the exposure (Cerdá-Moreno et al., 2024). Despite being a mild climate, the environmental conditions were suboptimal for the selected strain (winter/spring). It is important to highlight that this strain can be produced in this region during the whole year (Morillas-España et al., 2020).

**FIGURE 2:**
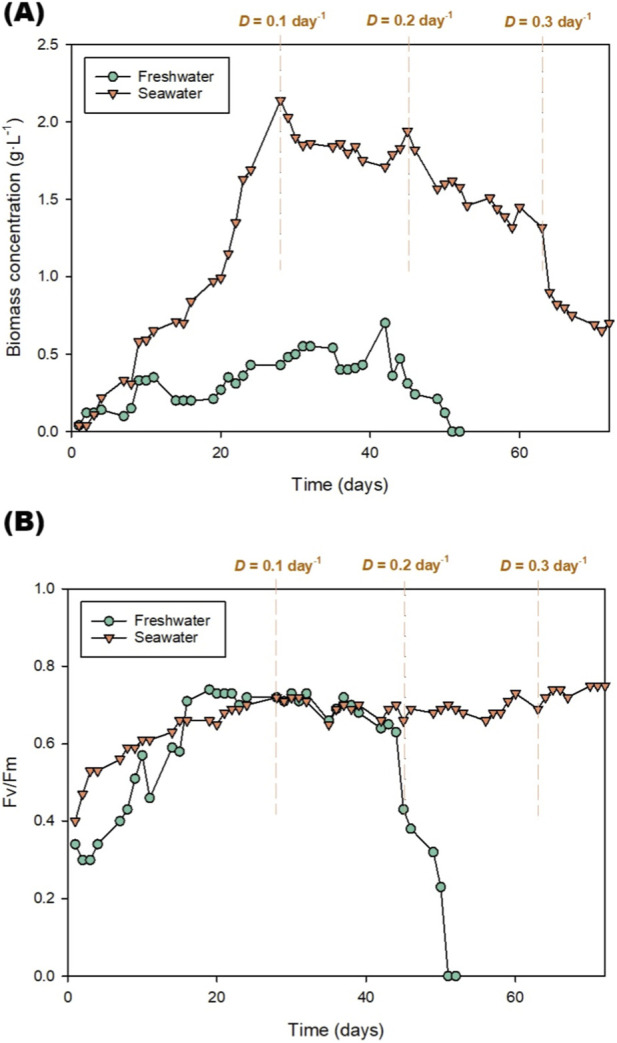
**(A)** Biomass concentration and **(B)** Fv/Fm value during biomass production using freshwater or seawater. Values represent the mean of three independent determinations. D refers to the dilution rate used during semi-continuous production. Values represent the mean of two independent photobioreactors.

The environmental conditions described above allowed the cells to grow as shown in Figure 2A, which displays the evolution of the biomass concentration in the culture. Both reactors were inoculated at a biomass concentration of approximately 0.1 g L^-1^. An adaptation period was observed during the first days in both cultures. The maximum concentration reached during the batch phase was remarkably different between both cultures; when produced using seawater, the biomass reached a biomass concentration of 2.2 g L^-1^, while it reached 0.6 g L^-1^ when produced using freshwater. The latter is a surprisingly low value as this microalga can be produced even in winter with higher productivities. The biomass was then produced in semi-continuous mode, working at a constant dilution rate of 0.1 day^-1^, which means that 10% of the culture volume was daily replaced with fresh culture medium. In the reactor operated using freshwater, the concentration remained at around 0.6 g L^-1^ while in the reactor operated using seawater it decreased slightly, ranging from 1.9 to 1.7 g L^-1^. The second dilution rate (0.2 days^-1^) was then applied, meaning that 20% of the culture was daily replaced with fresh culture medium. The freshwater culture crashed, meaning that the culture could not cope with such a high dilution rate. This microalga was produced using higher dilution rates in winter the past (Morillas-España et al., 2020). Therefore, the main hypothesis for the observed low productivity and the culture crash was the growth of an unwanted microorganisms. Indeed, microscopic observations (not shown) revealed the appearance of algal predators (e.g., rotifers) in the freshwater culture. This will be discussed in the next section of this study. In the reactor working with seawater, the concentration remained stable between 1.7 and 1.3 g L^-1^. Finally, a third dilution (0.3 days^-1^) was studied just in the seawater culture. In this case, the biomass concentration decreased from around 1.3–1.7 to 0.7 g L^-1^. Despite being a freshwater strain, the results showed that the biomass productivity was 4–5 times higher when using seawater compared to freshwater. In this study, the use of seawater favoured biomass growth. As highlighted before, the production of a freshwater strain using seawater leads to a high energetic consumption in osmoregulation and ion pumping that reduces biomass productivity. The results reported in this study contrast to previous work where seawater reduced biomass productivity (Villaró et al., 2023b). In this study, the main advantage observed was related to rotifers and other algal predators, which were not present in the culture produced using seawater (based on microscopic observations). It is known that high salinity inhibits halophobic fungi and rotifers, minimising the risk of population collapse and the need for costly anti-pollution treatments (Wang et al., 2024). However, this was not demonstrated for *Scenedesmus almeriensis* until this study.

### 3.2 Photosynthetic performance

Figure 2B shows the photochemical performance of *Scenedesmus almeriensis* under the different dilution regimes. The normal Fv/Fm value for *Scenedesmus almeriensis* growing in nutrient sufficient non-stressed cultures is around 0.5–0.7 (Morillas-España et al., 2020). In this study, both cultures started with low Fv/Fm values (0.30–0.35), characteristic of an adaptation period in industrial systems and for winter conditions. During the first week, the freshwater and seawater cultures had Fv/Fm values reaching 0.42 and 0.52, respectively. This indicated a faster acclimatisation of the seawater-adapted strain to the large reactor. Both cultures had Fv/Fm values of around 0.60–0.65 after a couple of weeks of production. This value remained constant during the rest of the biomass production and even increased slightly in the seawater culture once the semi-continuous phase started. The reason for this is that, during the semi-continuous production, part of the culture was daily replaced with fresh culture medium leading to higher nutrient and light availability. In the case of the seawater culture, the Fv/Fm values correlated well with those of biomass concentration. However, in the freshwater culture the biomass concentration was very low compared to that expected based on Fv/Fm determinations. This was not the case after operating the reactor at a dilution rate of 0.2 days^-1^, where both the biomass concentration and the Fv/Fm value decreased sharply. In turn, the Fv/Fm value of the seawater culture remained between 0.67 and 0.75, suggesting that the lower cell concentration (higher dilution rate) allowed greater light penetration and a nutrient supply, favouring photosynthetic activity. The higher stability of the seawater culture can also be attributed to the ionic matrix, which continuously provides macro and micronutrients and promotes the accumulation of osmoprotectants and antioxidants induced by salt stress.

**FIGURE 3:**
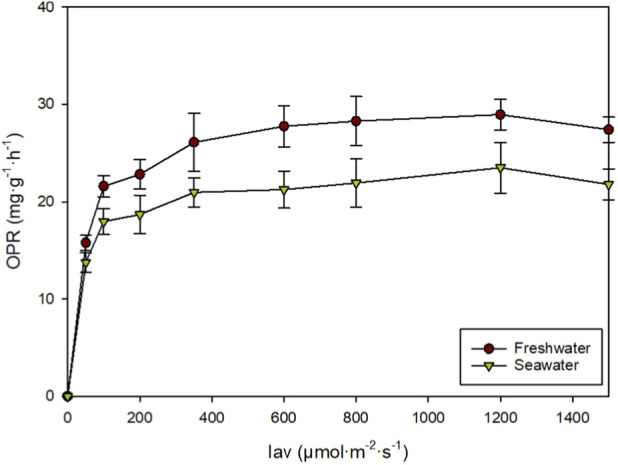
Oxygen production rate of *Scenedesmus almeriensis* cultured in freshwater and seawater under varying irradiance levels.

**FIGURE 4:**
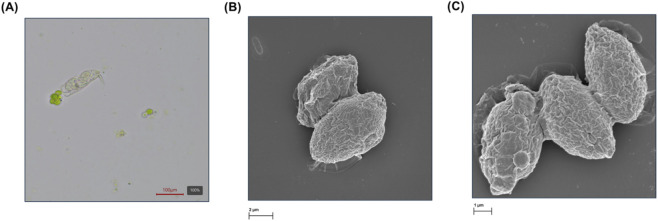
**(A)** Microscopic identification of an algal predator in the freshwater culture, **(B)** FESEM micrograph of the culture produced using freshwater and **(C)** FESEM micrograph of the culture produced using seawater.

While Fv/Fm provides an indicator of the maximum quantum yield of Photosystem II, the actual metabolic performance and oxygen evolution rates under the freshwater and seawater media were further characterised via photorespirometry. The OPR was determined for the freshwater and seawater strains (Figure 3). The results showed from 0 to 200 μmol m^-2^·s^-1^ a marked increase in the OPR in both samples attributed to a higher light availability. The cultures produced using freshwater or seawater reached maximum OPR values of around 29 and 24 mg g^-1^·h^-1^, respectively. Differences in photosynthetic efficiency could be attributed to the energy demand associated with osmotic regulation. In seawater cultures, part of the energy was dedicated to maintaining ionic homeostasis, which reduced the light energy available for CO_2_ fixation and O_2_ evolution (Aditi et al., 2025). In contrast, in freshwater, the osmotic load was lower, so a greater proportion of absorbed photons were allocated to light-dependent reactions and the Calvin cycle (Masojídek et al., 2021). This energy redistribution is reflected in the initial slope of the curve and in the OPR in freshwater cultures. These results show that microalgae produced with freshwater have significantly higher photosynthetic performance than those produced with seawater. This was expected as this microalga is a freshwater strain. However, this contrasts with the biomass concentration values discussed above. The paradox of higher productivity in seawater despite the lower OPR values can be explained by the catastrophic impact of external biological factors in the freshwater system (unwanted microorganisms degrading the culture; Figure 4A). The appearance of algal predators that can lead to a culture crush is common in pilot- and large-scale production facilities where environmental stressors (in this case, seawater) or physical barriers (e.g., greenhouses) are often required to prevent the proliferation of algal predators and ensure culture stability.

### 3.3 Microbial diversity

The results discussed in the previous sections demonstrated that despite higher photosynthetic performance, the culture produced using freshwater crashed during the semi-continuous production stage. Microscopic observations revealed the presence of rotifers and biotic contaminants were the most likely reason for the culture crash. Therefore, the microbial diversity of the cultures at the end of the batch phase was determined by means of microscopy and metabolomic analysis. Figures 4B,C show the morphology of the *Scenedesmus almeriensis* produced using freshwater and seawater, respectively. FESEM results show an irregular and elongated cell outline, with microreliefs and roughness attributed to the wall structure, composed of cellulose, alganins, and glycoproteins (Sankaranarayanan et al., 2018; Turiel et al., 2021; Zambrano et al., 2021). In both cultures, the morphological traits of the most abundant cells were within the typical range of 2–10 µm described for *Scenedesmus* strains (Jothibasu et al., 2022). Overall, FESEM results (not shown) revealed the dominance of carbon, oxygen, and nitrogen in the microalgal biomasses, with minor changes in inorganic elements, probably caused by the use of seawater instead of freshwater in one of the cultures. Based on the microscopic observations, a *Scenedesmus* strain was predominant in both cultures. However, the presence of rotifers in the freshwater-based culture was clear. In Figure 4A a specimen of the genus *Brachionus*, identified by its ciliated crown and trophomuscular apparatus, retaining numerous greenish cells in its digestive tract can be seen. This zooplanktonic infestation severely degrades the quality of the culture, as rotifers such as *Brachionus calyciflorus* can consume hundreds of algal cells per hour (Zhu et al., 2020). According to recent models, an infestation of rotifers in closed photobioreactors results in a total loss of productivity within days (Deruyck et al., 2019), as it occurred in this study.

**FIGURE 5:**
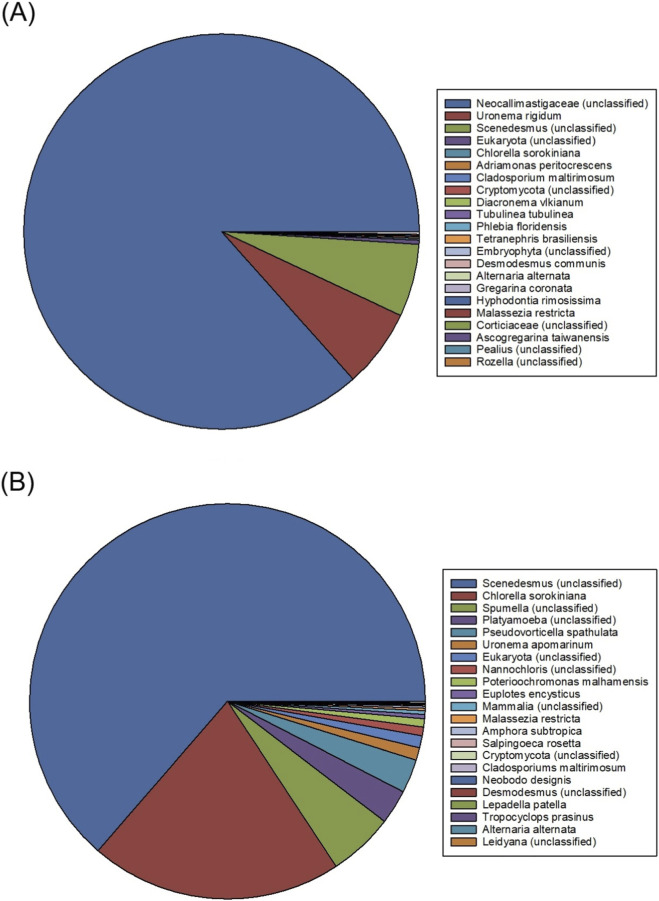
Taxonomic classification of microbial communities (18S) identified in the biomass produced using **(A)** freshwater and **(B)** seawater.

The presence of rotifers (and other biotic contaminants) was also evidenced by the metagenomic results. Figure 5A shows that up to 80% of the relative abundance (18S) in the freshwater culture were unidentified microorganisms of a taxon of the *Neocallimastigaceae* family, followed by *Uronema* (10%) and *Scenedesmus* (8%). The purity of the *Scenedesmus* culture was therefore low. The *Neocallimastigaceae* are anaerobic fungi belonging to the phylum *Neocallimastigomycota* (Lockhart et al., 2006). They are typically found in freshwater environments and can form fungal biofilms that agglomerate the algal cells (Li et al., 2021). This generates anoxic micro-zones and accumulation of dissolved organic matter. This phenomenon also reduces microalgal biomass productivity and causes cell wall weakening (Naseema Rasheed et al., 2023) further leading to cell lysis (Leis et al., 2014; Lillington et al., 2021; Stabel et al., 2021). This might be the main cause of the collapse of the culture being produced using freshwater described above. In addition, the genus *Uronema* (ciliate protozoa belonging to the class *Oligohymenophorea*) (Liu et al., 2017) also contributed to community imbalance through the phagocytosis of bacteria and, under scarce conditions, microalgae with a diameter of less than 4 µm (Schaafsma and Peperzak, 2013). Its rapid growth leads to direct competition for essential inorganic nutrients such as nitrogen and phosphorus, contributing to the decrease in biomass density (Band Schmidt, 2007). Their small size (20–50 µm) makes it difficult to remove by filtration or sedimentation, which favours their persistence and displacement of the target microalgae (Thomas et al., 2023). Studies on *Scenedesmus acuminatus* cultures correlated increased microzooplankton diversity (including *Uronema*) with reduced biomass yields (He et al., 2024). A culture of *Dunaliella salina* also collapsed in a previous study after 2 days with an increase in the concentration of ciliates (Moreno-Garrido and Cañavate, 2001). To mitigate these effects, control strategies that involve raising the pH level above 10 or adding 10 mg L^-1^ of sodium hypochlorite have been tested. These strategies have been shown to inhibit up to 95% of ciliates in *Chlorella vulgaris* cultures and 65% in *Scenedesmus acuminatus* cultures (Wang et al., 2017). The main limitation of these contingency measures is the difficulty of identifying the biotic contaminants in a fast way. Metabolomic studies are expensive and slow. Online sensors able to detect these contaminants are needed urgently at the industrial scale. Figure 5B lists the relative abundance (18S) of microorganisms in the seawater-based culture. In this case, the culture consisted mainly of *Scenedesmus* (70%) followed by *Chlorella sorokiniana* (18%) and *Spumella spp*. (8%). *Platyamoeba spp.,* and *Pseudovorticella spp*., were also identified but at significantly minor proportions. This indicates that the saline environment strongly limits common heterotrophic competitors in freshwater, favouring the dominance of the desired algae. It is important to highlight that a seawater strain of *Chlorella sorokiniana* was being produced simultaneously in an identical third photobioreactor located inside the same greenhouse. Airborne transport of microorganisms has been previously demonstrated (Huang et al., 2024). Bioaerosols generated in the bubble column of this third tubular reactor might have been the source of this biotic contamination as the water inlet and outlet piping was independent in both systems.

**FIGURE 6:**
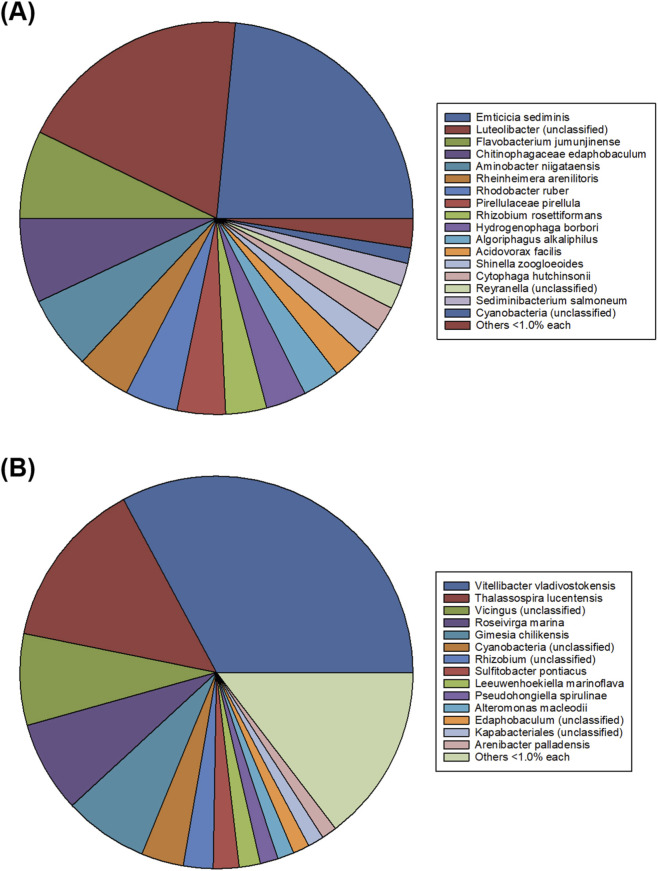
Taxonomic classification of microbial communities (16S) identified in the biomass produced using **(A)** freshwater and **(B)** seawater.

The microbial diversity of prokaryotic microorganisms in the freshwater and seawater cultures is shown in Figures 6A, B, respectively. The diversity was greater in the freshwater culture; *Emticicia sediminis* (25%), *Luteolibacter* (18%), *Flavobacterium jumunjinense* (8%), and *Chitinophagaceae edaphobaculum* (6%) account for the majority of the bacterial community, together with various genera of Proteobacteria and Bacteroidetes. This microbial diversity leads to intense competition for dissolved nutrients and available light, which limits microalgal growth and alters its physiology and metabolism (Yao et al., 2019). However, bacteria might have a positive impact on microalgal growth. Genera such as *Flavobacterium* degrade the polysaccharides and exopolysaccharides released by microalgae, favouring nutrient mineralisation and improving water quality (Thomas et al., 2017). Figure 6B shows that, under high-salinity conditions, the bacterial community was dominated by *Flavobacterium vladivostokensis* (24%), *Thalassospira lucentensis* (16%), *Vicingus* (unclassified) (9%), *Roseivirga marina* (8%) and, to a lesser extent, an unclassified cyanobacteria. In this environment, only halophilic or halotolerant bacteria coexist synergistically with the microalga, minimising competition for essential nutrients (Yao et al., 2019). For instance, it has been reported that *Thalassospira*, an *Alphaproteobacterium halophilus* facilitate algal growth under iron-limited conditions by producing chelating compounds or enhancing the uptake of this micronutrient (Abd-El-Aziz et al., 2025). Studies with *Chlorella sorokiniana* have demonstrated that microbial consortia increase resistance to invasion by harmful cyanobacteria, such as *Microcystis aeruginosa* (compared to an axenic culture) (Schmidt et al., 2020). Indeed, the symbiotic interaction between bacteria and microalgae in the phycosphere is known to optimise resource use and reduce reliance on external nutrient supplementation (Yao et al., 2019).

Overall, producing microalgae using seawater represents an environmental barrier against many biological contaminants. The high salt content inhibited freshwater bacteria, fungi, and protozoa that dominated the freshwater culture. In our case, no significant sequences of *Neocallimastigaceae* or other unwanted heterotrophs, which dominated the freshwater culture, were detected in the seawater culture. Several studies indicated that culture environments with extreme conditions allow longer monoclonal cultures to be maintained without significant interruption (Olofsson et al., 2019). In addition to its effect on biotic contaminants, the use of seawater significantly improves the sustainability of the process. Given the global scarcity of freshwater (the UN forecasts water stress in most countries by 2025), replacing freshwater-based processes with seawater reduces costs and frees up valuable water resources (Hirooka et al., 2020). Studies suggest that the use of saltwater on an industrial scale of algal cultivation could make a significant contribution towards economic and environmental viability (Arora et al., 2019; Hirooka et al., 2020). In addition, despite influencing biomass composition, a recent study revealed that the freshwater strain *Arthrospira platensis* can be produced using just seawater and that the resulting biomass meets the necessary conditions for food applications (Villaró et al., 2023b).

## 4 Conclusion

This study demonstrates that seawater is a viable and advantageous medium for the cultivation of *Scenedesmus almeriensis* at pilot scale. Compared to freshwater, seawater-based cultures achieved significantly higher biomass productivity and exhibited greater resistance to microbial contamination. The saline environment acted as a biological barrier, reducing the presence of harmful organisms such as rotifers and fungi, which severely impacted freshwater cultures. Furthermore, seawater supported a more stable and synergistic microbial community, enhancing nutrient recycling and promoting algal growth. These results support the integration of seawater into industrial microalgae cultivation systems as a sustainable strategy to reduce freshwater consumption and improve overall process performance. In this case, the microalga produced was a freshwater strain that was gradually acclimated to seawater, highlighting the potential for using seawater not just in marine strains.

## Data Availability

The raw data supporting the conclusions of this article will be made available by the authors, without undue reservation.
